# Practicality of identifying mitochondria variants from exome and RNAseq data

**DOI:** 10.1186/1471-2105-16-S15-P6

**Published:** 2015-10-23

**Authors:** Pan Zhang, David C Samuels, Brian Lehmann, Thomas Stricker, Jennifer Pietenpol, Yu Shyr, Yan Guo

**Affiliations:** 1 Center for Quantitative Sciences, Vanderbilt University, Nashville TN 37027, USA; 2 Center for Human Genetics Research, Vanderbilt University, Nashville TN 37037, USA; 3 Department of Biochemistry, Vanderbilt University, Nashville TN 37232, USA; 4 Department of Pathology, Vanderbilt University, Nashville TN 37027, USA

## Background

The rapid progress in high throughput sequencing technology has significantly enriched our capability to study mitochondria genomes. Other than performing mitochondria targeted sequencing, an increasingly popular alternative approach is to utilize the off-target reads from exome sequencing to infer mitochondria genomic variants including SNP and heteroplasmy[1-9]. However, the effectiveness and practicality of such an approach has not been tested. Recently, RNAseq data has also been suggested as good source for alternative data mining[10,11], but whether mitochondria variants are minable has not been studied.

## Materials and methods

We designed a specific study using targeted mitochondria sequencing data as a gold standard to evaluate the practicality of SNP and heteroplasmy detection using exome sequencing and RNAseq data. Five breast cancer cell lines were sequenced for mitochondria targeted sequencing, exome sequencing, and RNAseq. Furthermore, we examined three mitochondria alignment strategies: 1) align all reads directly to the mitochondria genome; 2) align all reads to the nuclear genome and mitochondria genome simultaneously; 3) align all reads to the nuclear genome first, then used the unaligned reads to align to the mitochondria genome.

## Results

Our analyses found that exome sequencing can accurately detect mitochondria SNPs and can detect a portion of the true heteroplasmies with a reasonable false discovery rate. RNAseq data on the other hand had a lower detection rate of SNP but higher detection rate for heteroplasmy. However, the higher false discovery rate makes RNAseq a less ideal source for studying mitochondria compared to exome sequencing data. Furthermore, we found that aligning all reads directly to the mitochondria genome reference or aligning all reads to the nuclear genome and mitochondria genome references simultaneously produced the best results.

## Conclusions

Exome sequencing and RNAseq data can be potentially mined for mitochondria variants. Overall, exome sequencing provides less false discovery than RNAseq for mitochondria variant detection, making it a more desirable choice. In conclusion, our study provides important guidelines for future studies that intend to use exome sequencing or RNAseq data to infer mitochondria SNP and heteroplasmy.
